# The Diagnostic Yield of the Multidisciplinary Discussion in Patients With COVID-19 Pneumonia

**DOI:** 10.3389/fmed.2021.637872

**Published:** 2021-04-01

**Authors:** Fiorella Calabrese, Federica Pezzuto, Chiara Giraudo, Luca Vedovelli, Francesco Fortarezza, Claudia Del Vecchio, Francesca Lunardi, Anna Sara Fraia, Elisabetta Cocconcelli, Stefania Edith Vuljan, Dario Gregori, Andrea Crisanti, Elisabetta Balestro, Paolo Spagnolo

**Affiliations:** ^1^Department of Cardiac, Thoracic, Vascular Sciences, and Public Health, University of Padova, Medical School, Padova, Italy; ^2^Department of Medicine, University of Padova, Medical School, Padova, Italy; ^3^Department of Molecular Medicine, University of Padova, Medical School, Padova, Italy

**Keywords:** diagnostic yield, multidisciplinary approach, COVID-19, COVID-19 pneumonia, SARS-CoV-2

## Abstract

**Purpose:** The hypothesis of the study was that a multidisciplinary approach involving experienced specialists in diffuse parenchymal lung disease might improve the diagnosis of patients with COVID-19 pneumonia.

**Methods:** Two pulmonologists, two radiologists, and two pathologists reviewed 27 patients affected by severe COVID-19 pneumonia as the main diagnosis made by non-pulmonologists. To evaluate whether the contribution of specialists, individually and/or in combination, might modify the original diagnosis, a three-step virtual process was planned. The whole lung examination was considered the gold standard for the final diagnosis. The probability of a correct diagnosis was calculated using a model based on generalized estimating equations. The effectiveness of a multidisciplinary diagnosis was obtained by comparing diagnoses made by experienced pulmonologists with those made by non-pulmonologists.

**Results:** In 19% of cases, the diagnosis of COVID-19-related death was mainly incorrect. The probability of a correct diagnosis increased strikingly from an undedicated clinician to an expert specialist. Every single specialist made significantly more correct diagnoses than any non-pulmonologist. The highest level of accuracy was achieved by the combination of 3 expert specialists (*p* = 0.0003).

**Conclusion:** The dynamic interaction between expert specialists may significantly improve the diagnostic confidence and management of patients with COVID-19 pneumonia.

## Introduction

Coronavirus disease 19 (COVID-19) was first identified in Wuhan, China, in December 2019 and is now on its second wave. Genetic sequencing of the virus determined that it is a beta coronavirus named severe acute respiratory syndrome-related coronavirus 2 (SARS-CoV-2) (1). Although most patients have a favorable prognosis, pneumonia, and severe hypoxemia secondary to SARS-CoV-2 infection can lead to acute respiratory failure (ARF) and death (2). Elderly male patients with comorbidities such as obesity, hypertension, diabetes, cardiac disease, and neoplasm also have an increased risk for severe disease and death and need distinct management and higher surveillance levels (3–6). Management remains suboptimal with high mortality rates, particularly among patients admitted to the intensive care unit (ICU).

The integration of all available data from each patient has proven crucial in the management of diffuse parenchymal lung disease (DPLD). Indeed, the international guidelines suggest health professionals with experience in DPLD be involved in patient diagnosis and management in a multidisciplinary approach to achieve the most confident diagnosis and optimize treatment (7). The sudden onset and rapid spread of the COVID-19 pandemic with the high number of infections and deaths have led to a global health emergency. Since this was an unknown disease, the priority has been stemming the infection, which inevitably has limited interaction among experts. As COVID-19 is an extremely complex disease, it would potentially benefit from a multidisciplinary approach even during the pandemic. The hypothesis of this study was therefore that a multidisciplinary approach involving specialists experienced in DPLD (pulmonologists, radiologists, and pathologists) may improve the diagnosis and management of patients with COVID-19 pneumonia, following a decision-making approach similar to what is used in DPLD.

## Materials and Methods

### Study Subjects

The present study was a critical re-evaluation of deceased patients by an expert team of specialists (pulmonologists, radiologists, and pathologists) routinely involved in multidisciplinary meetings of mainly DPLD (8) but also with robust experience in COVID-19 diagnosis and management (9, 10). We retrospectively studied 27 patients who consecutively died from March to May 2020 in our hospital whose death certificate indicated SARS-CoV-2 infection (detected at least once on nasopharyngeal swab) and severe COVID-19 pneumonia as the main diagnosis made by non-pulmonologists (i.e., emergency room clinicians, general practitioners, specialists in infectious diseases, and anaesthesiologists).

The autopsy was performed according to national and international protocols, as previously described (11). At autopsy, the whole lungs were macroscopically examined. A small sample was taken from the most representative area of lung injury. The sample was in part preserved in RNA later and processed for molecular analyses (see below for molecular processing details) and in part fixed in formalin for routine histology, before and independently from the other additional fragments. Pathological features suggestive of COVID-19 pneumonia (alveolar injury as well as vascular lesions) were quantitatively described using a scoring system, as previously reported (10). Other associated lesions (neoplasia, infectious diseases, aspiration pneumonia, etc.,) were also reported. To confirm the pathological diagnosis of COVID-19 pneumonia, the fragment preserved in RNA later was also processed by real time reverse transciptase-polymerase chain reaction (RT-PCR) for SARS-CoV-2 [SARS-CoV-2 (2019-nCoV) Centers for Disease Control and Prevention (CDC) Emergency Use Authorized (EUA) Authorized qPCR probe assay primer/probe mix]. An additional fragment was analyzed by culture. Briefly, virus isolation was performed using African green monkey kidney (Vero) cells. When a diffuse, refractile, rounding, cytopathic effect was noted, the Vero cell culture supernatant was passaged to a fresh Vero cell culture tube to ensure reproducibility of the cytopathic effect. SARS-CoV-2 in the supernatant was further confirmed by reverse transcription polymerase chain reaction using primers described previously (12).

In order to define the levels of certainty for a diagnosis of COVID-19 pneumonia, two expert pathologists (FC, FP) scored all cases independently and blinded to clinical and autopsy data. Several parameters, distinguishing distinct anatomic areas (airways, alveolar wall, alveolar space, pleura) and any additional findings were recorded, as previously described (13). Based on morphological/virologic evaluation, four distinct levels of diagnostic certainty were defined: (1) Definite COVID-19 pneumonia: all lung samples showing lesions typical of COVID-19 pneumonia (thrombosis, vascular injury, and/or diffuse alveolar damage/organizing pneumonia), confirmed SARS-CoV-2 lung positivity (both molecular and culture), with no other associated or pre-existing lesions (e.g., foci of bacterial pneumonia, neoplasia), (2) Probable COVID-19 pneumonia: lung samples displaying mainly features of COVID-19 pneumonia (+/– lung SARS-CoV-2 infection) with other associated lesions (e.g., foci of bacterial infection), (3) Possible COVID-19 pneumonia: lung samples showing only focal changes of COVID-19 pneumonia (+/– lung SARS-CoV-2 infection, etc.,) with more extensive features consistent with alternative diagnoses (i.e., lung cancer/metastasis, etc.,), (4) Non-COVID-19 pneumonia: lung samples not showing any typical lesions, no evidence of SARS-CoV2 infection, and presence of features consistent with alternative diagnoses.

During the autopsy, additional fragments were sampled from both lungs (at least 20 samples for each case) and systematically analyzed, as previously described (13, 14).

Clinical evaluation was performed by two experienced pulmonologists (PS, EB) based on the following data: past and recent medical history including comorbidities, respiratory and systemic signs, and symptoms (type and duration) before hospital admission, imaging, laboratory findings, gas exchange values (FiO2, pO2, and pO2/FiO2) and their changes during hospitalization, and oxygen supplementation. Based on this data, patients were classified as follows: (1) Definite COVID-19 pneumonia: clinical findings typical of COVID-19 such as severe acute respiratory illness (i.e., fever, cough, shortness of breath, hypoxemia) in the absence of an alternative diagnosis that could explain the clinical presentation (15, 16), (2) Probable COVID-19 pneumonia: features of COVID-19 pneumonia associated with findings suggestive of alternative diagnoses (e.g., pleural effusion, clinical and laboratory findings in keeping with heart failure, or signs of bacterial pneumonia), (3) Possible COVID-19 pneumonia: features of COVID-19 pneumonia associated with prevalent findings consistent with alternative etiologies (e.g., lung cancer, pulmonary metastases, pulmonary edema, heart failure), (4) Non-COVID-19 pneumonia: absence of typical signs/symptoms and laboratory findings of COVID-19 pneumonia in the presence of features consistent with alternative diagnoses (e.g., neoplasm, interstitial lung disease, ischemic heart disease, pulmonary edema).

With regard to the radiological assessment, all chest X-rays and, when available, chest computed tomography (CT) images were assessed by two expert thoracic radiologists (CG, AF). According to the radiological findings, patients were classified as follows: (1) Definite COVID-19 pneumonia: typical findings of COVID-19 pneumonia, such as bilateral ground-glass opacities and/or consolidations (17), without any signs of alternative diagnoses, (2) Probable COVID-19 pneumonia: features of COVID-19 pneumonia associated with abnormalities such as pleural effusion, cardiomegaly, or Kerley B lines suggestive of cardiac failure, or lobar consolidation suggestive of bacterial pneumonia, (3) Possible COVID-19 pneumonia: features of COVID-19 pneumonia associated with predominant findings of alternative diagnoses (e.g., unilateral pulmonary lesions due to lung cancer, pulmonary bilateral metastatic nodules), (4) Non-COVID-19 pneumonia: no typical signs of COVID-19 with features suggestive of alternative diagnoses (e.g., unilateral pulmonary lesions due to lung cancer, reticular changes secondary to interstitial lung disease).

Data regarding demographics, smoking history, symptoms, comorbidities, treatment, disease duration, serology, radiological, and pathological findings were included in a dedicated database in REDCap. Informed consent was granted by a relative/legal representative of each deceased patient. The study was approved by the local clinical institutional review Board.

### Study Designs

To evaluate whether the contribution of pulmonologists, radiologists, and pathologists individually and/or in combination, could change the diagnosis originally made by non-pulmonologists, we planned a three-step process, modifying the methodology previously used in the evaluation of patients with DPLD (18).

**Figure 1:**
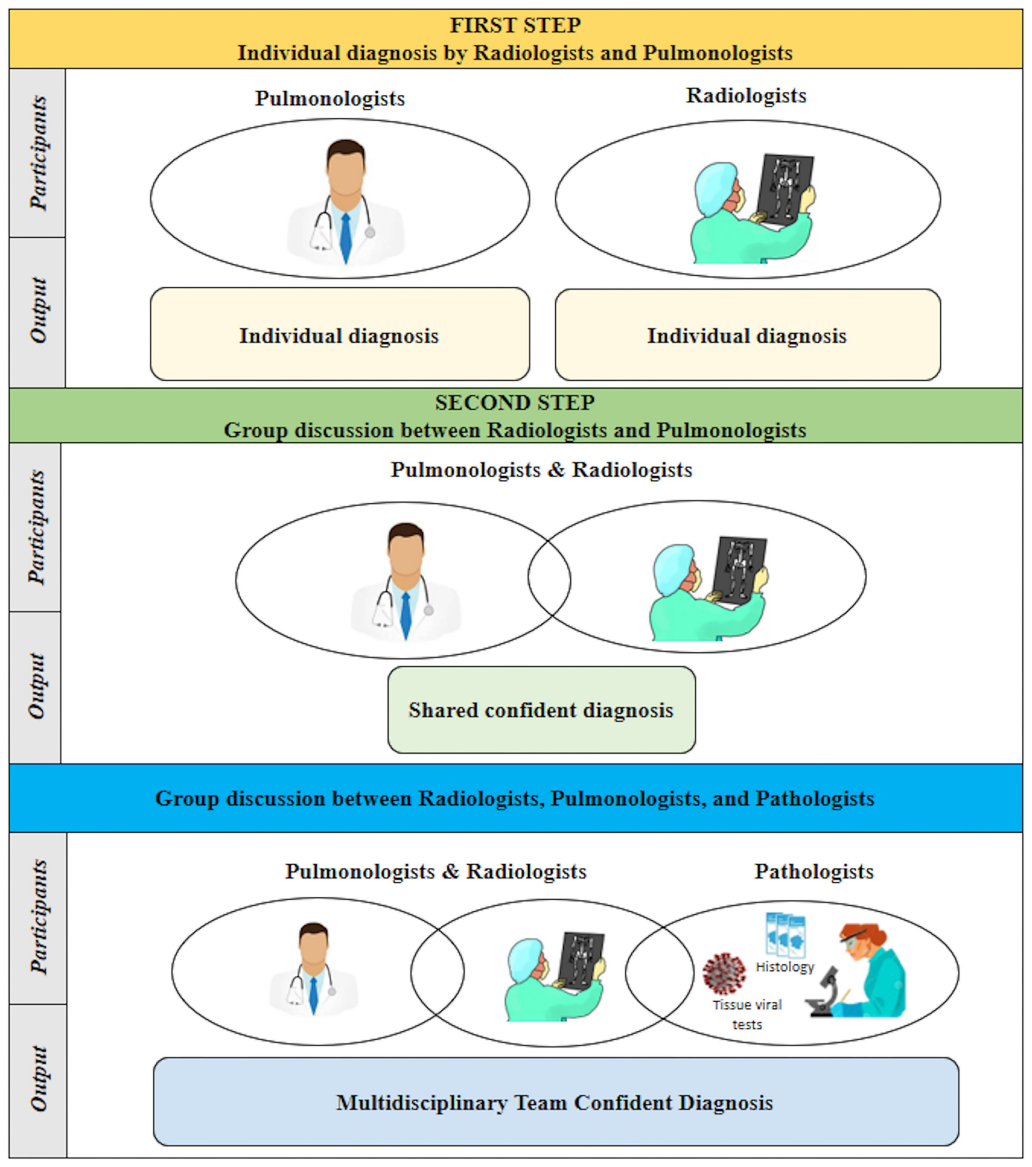
Graphic representation of multistep processing in the decision-making approach.

Briefly, in the first step, two pulmonologists (PS, EB) and two radiologists (CG, AF) independently reviewed clinical and radiological data for each patient, without pathological data, and recorded their individual diagnoses and confidence levels. In the second step, pulmonologists and radiologists discussed their diagnosis and again recorded their individual or shared (in case of disagreement) confidence level. During the third step, pathologists entered the arena and reported the pathological diagnosis performed on a single lung fragment. The final diagnosis derived from the whole lung examination and full organ autopsy and was considered the diagnostic gold standard. Virtual meetings via the Zoom platform were set to allow pulmonologists, radiologists, and pathologists to discuss their interpretation with mutual collaboration (Figure 1).

### Analysis

All patients were evaluated by pulmonologists and pathologists. One case was not evaluated by radiologists due to lack of radiological data. Specialist scores (for single specialist and for combinations of different specialists) were compared with the full autopsy diagnosis, that was considered to be the true diagnosis, and were recorded as “correct” or “wrong”. Probability of a correct diagnosis (95% confidence interval) was calculated with the method of Wilson using the binconf function of the R package {Hmisc} (19). To explore the effectiveness of a multidisciplinary diagnosis we compared specialist diagnoses with the non-pulmonologist using a model based on generalized estimating equations (GEE) (20) which expand the application of generalized linear models, providing a framework for analyzing correlated data, especially from repeated measures studies where multiple observations are collected from a specific sampling unit (21). In particular, we used a first-order autoregressive correlation structure and a robust standard error estimation to fit our small sample size. The R package {geepack} was used for the analysis (20). We exponentiated GEE results to obtain an odds ratio (95% CI) for each specialist (or combination of specialists) on their ability to formulate a correct diagnosis. All analyses and plotting were conducted on R software v.4.0.2 (22). The full code used for the analysis is available upon request.

## Results

### Study Population

For all patients, demographic, clinical, and laboratory data are summarized in Table 1.

**Table 1:** Main clinical, epidemiological, and laboratory data available for all patients.

**ID**	**Epidemiological data**				**SARS-COV-2-RT-PCR**	**Symptoms**			**Laboratory tests**				**Symptoms before admission**	**Ward**
	**Sex**	**Age yrs**	**Smoke**	Number of comorbidities^*^	**NF SWAB**	**Fever C°**	**Cough**	**Dyspnea**	**WBC 10^**9**^/L**	**LY 10^**9/**^L**	**LY %**	**D-D μg/L**	**Days**	
1	M	82	No	2	Positive	39.5	Yes	Yes	15.17	0.79	5.2	311	5	ICU
2	F	69	Na	2	Positive	37.9	No	Yes	8.94	1.09	12.2	2063	5	ICU
3	M	76	Na	3	Positive	38	No	Yes	9.14	1.02	7.9	736	3	ICU
4	M	71	No	1	Positive	38	Yes	No	17.98	1.69	9.4	684	14	ICU
5	F	87	Former	4	Positive	39	No	Yes	13.15	0.65	4.9	2929	3	Non-ICU
6	M	79	Yes	3	Positive	37.6	Yes	Yes	2.68	0.62	23.1	450	2	ICU
7	M	85	Former	5	Positive	38	Yes	Yes	18.09	0.79	4.4	772	4	Non-ICU
8	M	76	No	2	Positive	38	No	Yes	5.28	1.36	25.8	150	0	ICU
9	F	86	No	5	Positive	<37	No	Yes	12.16	0.83	6.8	na	5	Non-ICU
10	M	96	No	5	Positive	<37	No	Yes	4.99	1.63	32	150	2	Non-ICU
11	M	86	No	4	Positive	38.6	Yes	Yes	2.7	0.4	14.3	3250	3	Non-ICU
12	M	77	No	3	Positive	39	Yes	Yes	4.08	0.81	19.9	497	18	ICU
13	F	90	No	4	Positive	39	Yes	Yes	17.83	0.77	4.3	Na	7	Non-ICU
14	M	80	No	4	Positive	38.5	Yes	Yes	0.99	Na	Na	Na	2	Non-ICU
15	F	73	Na	1	Positive	37.8	No	Yes	5.52	0.67	12.2	345	2	ICU
16	M	61	Yes	3	Positive	<37	No	Yes	3.02	Na	Na	Na	5	ICU
17	M	82	Former	6	Positive	38	Yes	Yes	3.72	0.46	12.4	1711	3	Non-ICU
18	M	75	No	0	Positive	38.5	Yes	Yes	5.48	0.49	9.8	370	7	ICU
19	M	95	Former	3	Positive	<37	Yes	Yes	20.36	1.24	6.1	176	2	Non-ICU
20	F	88	No	5	Positive	38.5	Yes	Yes	6.45	0.94	14.6	251	4	Non-ICU
21	F	74	Former	3	Positive	38	Yes	Yes	5.2	0.8	15.4	150	10	ICU
22	F	87	No	3	Positive	37	Yes	Yes	2.6	0.49	18.6	644	8	Non-ICU
23	F	92	Na	2	Positive	38.5	No	No	16.37	0.95	5.8	1660	2	Non-ICU
24	F	90	Na	3	Positive	37.7	Yes	Yes	Na	Na	Na	Na	1	Non-ICU
25	M	83	Former	1	Positive	<37	Yes	Yes	5.78	0.61	10.6	Na	6	Non-ICU
26	F	97	Na	3	Positive	37.9	Yes	No	7.37	1.49	75.4	Na	0	Non-ICU
27	F	42	Na	1	Positive	<37	No	Yes	4.32	0.70	16.2	Na	33	Non-ICU

*Yrs, years; na, not available; WBC, white blood cells; Ly, lymphocytes; D-D, d-dimer; ICU, intensive care unit; RT, reverse transcriptase; PCR, polymerase chain reaction; NF, nasopharyngeal*.

* *List of the most common comorbidities detected: Arterial hypertension, cardiovascular diseases (atrial fibrillation, valvular heart disease, cardiac failure, vasculopathy, angiodysplasia, chronic cerebral vasculopathy, pulmonary embolism, aortic aneurysm), kidney diseases (chronic renal failure, kidney transplant), chronic conditions (diabetes, dyslipidemia, dementia, chronic obstructive pulmonary disease, hyperthyroidism, and connective tissue diseases)*.

The patient population included 15 males (56%) and 12 females (44%) with a median age of 82 years (overall range 42–97 years, interquartile range, 75.5–87.5 years). At disease onset, the main common complaints were dyspnea (89%), fever (74%), and cough (67%). On admission, white blood cells (WBC), and lymphocytes showed a median value of 2 × 10^9^/L (overall range 0.99–20.36 × 10^9^/L, interquartile range 4.14–12.90 × 10^9^/L) and 0.79 × 10^9^/L (overall range 0.4–1.69 × 10^9^/L, interquartile range 0.6425–1.0375 × 10^9^/L), respectively. D-dimer levels were available for 19 patients, with a median value of 497 μg/L (overall range 150–3250 μg/L, interquartile range 281–1216 μg/L). Overall, the radiologists assessed 97 chest X-rays and four CTs. On average, for each patient four chest X-rays were available.

### Multistep Process and Interobserver Agreement

During the first step (Individual diagnosis by Radiologists and Pulmonologists), pulmonologists categorized 11 cases as definite (41%), nine cases as probable (33%), three cases as possible (11%), and four as non-COVID-19 pneumonia (15%). Radiologists classified 11 patients as definite (42%), 11 patients as probable (42%), two patients as possible (8%) and two patients as non-COVID-19 pneumonia (8%). The radiological data of one patient was not available. The overall diagnoses with their corresponding level of confidence are reported in Table 2.

**Table 2:** Confident diagnoses achieved step by step.

**ID**	**First step**		**Second step**			**Third step**				
	**Individual diagnosis of pulmonologists and radiologists**		**Group discussion between pulmonologists and radiologists**			**Group discussion between pulmonologists, radiologists, and pathologists**				
	**Pulmonologists**	**Radiologists**	**Pulmonologists**	**Radiologists**	**Agreement**	**Pulmonologists + Radiologists**	**Pathologists**	**Agreement**	**MDT confident diagnosis**	**Final diagnosis (after the evaluation of all lung specimens and full autopsy)**
	**Output**	**Output**	**Output**	**Output**		**Output**	**Output**			
1	Definite	Definite	Definite	Definite	Yes	Definite	Definite	Yes	Definite	COVID-19 pneumonia
2	Definite	Definite	Definite	Definite	Yes	Definite	Definite	Yes	Definite	COVID-19 pneumonia
3	Definite	Definite	Definite	Definite	Yes	Definite	Definite	Yes	Definite	COVID-19 pneumonia
4	Other	Probable	Other	Probable	No	Other/Probable	Other	Yes, partial	Other	Iatrogenic pneumonia
5	Probable	Probable	Probable	Probable	Yes	Probable	Definite	No	Probable	COVID-19 pneumonia & edema
6	Definite	Definite	Definite	Definite	Yes	Definite	Definite	Yes	Definite	COVID-19 pneumonia
7	Other	Probable	Other	Probable	No	Other/Probable	Probable	Yes, partial	Probable	COVID-19 pneumonia & squamous cell carcinoma
8	Definite	Definite	Definite	Definite	Yes	Definite	Definite	Yes	Definite	COVID-19 pneumonia
9	Other	Other	Other	Other	Yes	Other	Probable	No	Probable	COVID-19 pneumonia & bacterial pneumonia
10	Other	Probable	Other	Probable	No	Other/Probable	Other	Yes, partial	Other	COVID-19 pneumonia & bacterial pneumonia
11	Probable	Definite	Probable	Definite → Prob	Yes	Probable	Definite	No	Definite	COVID-19 pneumonia
12	Possible	Possible	Possible	Possible	Yes	Possible	Possible	Yes	Possible	Squamous cell carcinoma & foci of COVID-19 pneumonia
13	Definite	Probable	Definite → Prob	Probable	Yes	Probable	Definite	No	Definite	COVID-19 pneumonia
14	Probable	Probable	Probable	Probable	Yes	Probable	Probable	Yes	Probable	COVID-19 pneumonia & bacterial pneumonia
15	Possible	Possible	Possible	Possible	Yes	Possible	Possible	Yes	Possible	Malignant SFT & foci of COVID-19 pneumonia
16	Probable	Probable	Probable	Probable	Yes	Probable	Probable	Yes	Probable	COVID-19 pneumonia & Aspergillus bronchopneumonia
17	Probable	Probable	Probable	Probable	Yes	Probable	Probable	Yes	Probable	COVID-19 pneumonia & bacterial pneumonia
18	Definite	Definite	Definite	Definite	Yes	Definite	Definite	Yes	Definite	COVID-19 pneumonia
19	Definite	Other	Definite → Other	Other	Yes	Other	Other	Yes	Other	Aspiration and bacterial pneumonia
20	Probable	Probable	Probable	Probable	Yes	Probable	Probable	Yes	Probable	COVID-19 pneumonia & necrotizing granulomas
21	Definite	Definite	Definite	Definite	Yes	Definite	Definite	Yes	Definite	COVID-19 pneumonia
22	Probable	Definite	Probable	Definite → Prob	Yes	Probable	Probable	Yes	Probable	COVID-19 pneumonia & aspiration pneumonia
23	Probable	Definite	Probable	Definite → Prob	Yes	Probable	Probable	Yes	Probable	COVID-19 pneumonia & bacterial pneumonia
24	Definite	Na	Definite	Na	Na	Na	Possible	Na	Possible	Bacterial pneumonia & foci of COVID-19 pneumonia
25	Possible	Probable	Possible → Prob	Probable	Yes	Probable	Definite	No	Definite	COVID-19 pneumonia
26	Definite	Definite	Definite	Definite	Yes	Definite	Definite	Yes	Definite	COVID-19 pneumonia
27	Probable	Probable	Probable	Probable	Yes	Probable	Probable	Yes	Probable	COVID-19 pneumonia & breast cancer metastasis

*na, not available; MDT, multidisciplinary team; Prob, probable; SFT, solitary fibrous tumor*.

During the second step (Discussion between Radiologists and Pulmonologists), a confident diagnosis was reached in 23 out of 26 cases (88%) with definite COVID-19 pneumonia in eight cases (30%). Following discussion, the diagnosis was changed in six cases, three changes for each specialist group (changes indicated with arrows in Table 2).

In the third step (Group discussion involving Radiologists, Pulmonologists, and Pathologists), the pathologists reported a diagnosis of definite COVID-19 pneumonia in 12 cases (45%), probable COVID-19 pneumonia in nine cases (33%), and possible COVID-19 pneumonia in three cases (11%). Three cases were classified as non-COVID-19 pneumonia (11%) (Table 3). A multidisciplinary discussion led to a confident diagnosis in 18 cases (69%), a partial agreement in three cases (12%), and no agreement in five cases (19%) (Table 2).

**Table 3:** Results of lung histological examination, molecular tissue and cultural analyses.

**ID**	**Histological findings**		**Molecular tests**		**Pathological diagnosis**
	COVID-19 related lesions^*^	**Other lesions**	**Tissue real time RT-PCR**	**Culture**	
1	Diffuse alveolar damage/organizing pneumonia and vascular injury		+	+	Definite
2	Diffuse alveolar damage/organizing pneumonia and vascular injury		+	Na	Definite
3	Diffuse alveolar damage/organizing pneumonia and vascular injury		+	+	Definite
4	No lesions	Iatrogenic paracetamol injury	–	Na	Other
5	Diffuse alveolar damage/organizing pneumonia		+	+	Definite
6	Diffuse alveolar damage/organizing pneumonia		+	+	Definite
7	Multiple foci of diffuse alveolar damage/organizing pneumonia and vascular injury	Bronchial squamous cell carcinoma	+	+	Probable
8	Diffuse alveolar damage/organizing pneumonia		+	Na	Definite
9	Multiple foci of diffuse alveolar damage/organizing pneumonia and vascular injury	Bacterial pneumonia	–	Na	Probable
10	No lesions	Bacterial pneumonia	+	Na	Other
11	Diffuse alveolar damage/organizing pneumonia and vascular injury		+	Na	Definite
12	Foci of diffuse alveolar damage/organizing pneumonia and vascular injury	Diffuse squamous cell carcinoma	+	Na	Possible
13	Diffuse alveolar damage/organizing pneumonia and vascular injury		+	Na	Definite
14	Multiple foci of diffuse alveolar damage/organizing pneumonia and vascular injury	Bacterial pneumonia	+	+	Probable
15	Foci of diffuse alveolar damage/organizing pneumonia and vascular injury	Malignant pleural solitary fibrous tumor	+	–	Possible
16	Multiple foci of diffuse alveolar damage/organizing pneumonia and vascular injury	Aspergillus invasive bronchopneumonia	+	+	Probable
17	Multiple foci of diffuse alveolar damage/organizing pneumonia and vascular injury	Bacterial pneumonia	+	–	Probable
18	Diffuse alveolar damage/organizing pneumonia and vascular injury		+	–	Definite
19	No lesions	Aspiration/bacterial pneumonia	–	–	Other
20	Multiple foci of diffuse alveolar damage/organizing pneumonia and vascular injury	Necrotizing granulomas	+	+	Probable
21	Diffuse alveolar damage/organizing pneumonia		+	–	Definite
22	Multiple foci of diffuse alveolar damage/organizing pneumonia and vascular injury	Aspiration pneumonia	+	+	Probable
23	Multiple foci of diffuse alveolar damage/organizing pneumonia and vascular injury	Bacterial pneumonia	+	–	Probable
24	Foci of diffuse alveolar damage/organizing pneumonia and vascular injury	Bacterial pneumonia	+	–	Possible
25	Diffuse alveolar damage/organizing pneumonia and vascular injury		+	–	Definite
26	Diffuse alveolar damage/organizing pneumonia and vascular injury		+	+	Definite
27	Multiple foci of diffuse alveolar damage/organizing pneumonia and vascular injury	Breast cancer metastases/bacterial pneumonia	+	–	Probable

*RT, reverse transcriptase; PCR, polymerase chain reaction; na: not available*.

* *List of the most common histological findings detected. Diffuse alveolar damage/organizing pneumonia: hyaline membrane, edema, and hemorrhage (acute exudative phase); type 2 pneumocyte hyperplasia, organizing pneumonia, and squamous metaplasia (organizing/proliferative phase). Vascular injury: capillary inflammation, neutrophilic capillaritis, microthrombi and macrothrombi*.

**Figure 2:**
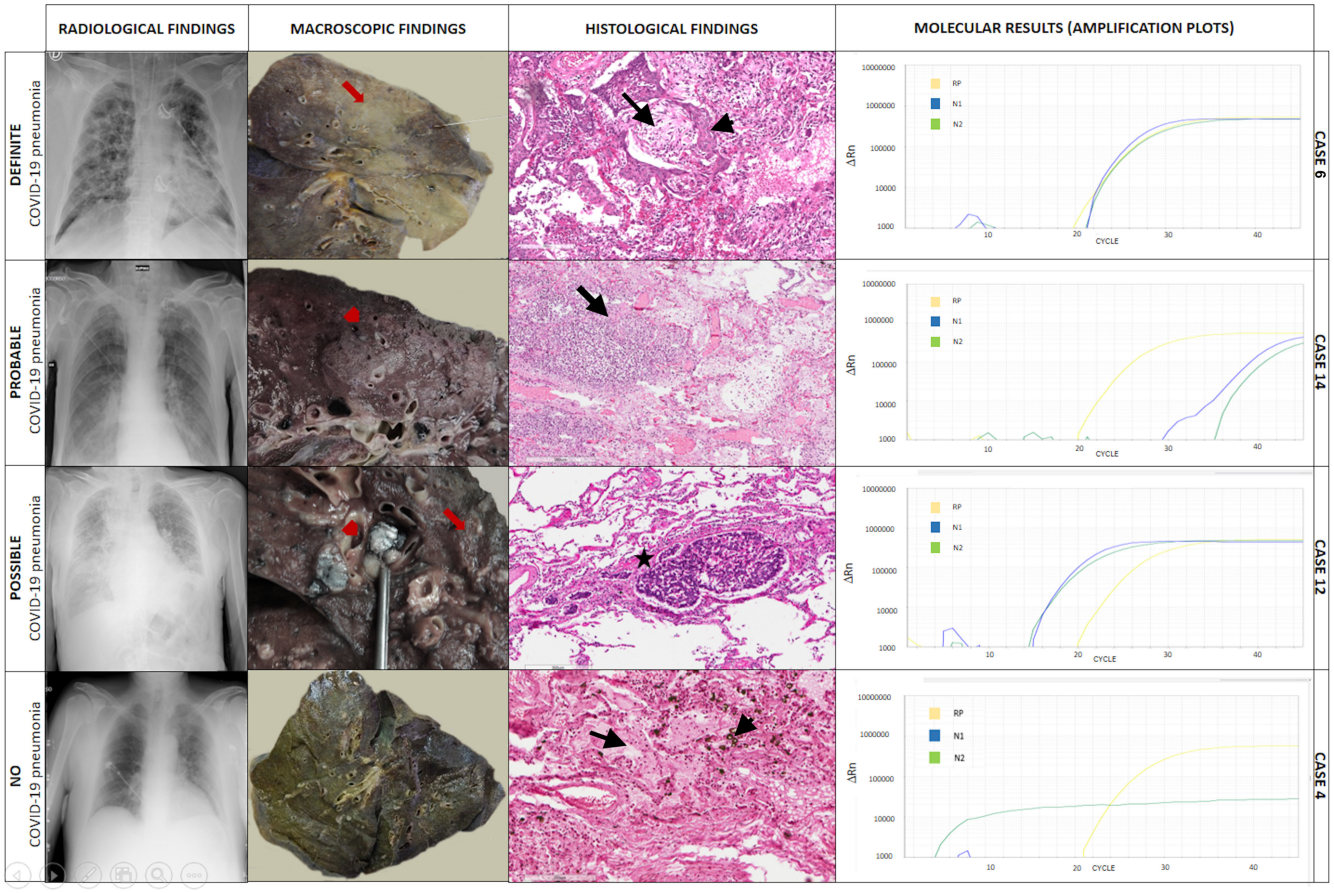
Explicative cases of definite, probable, possible, and non-COVID-19 pneumonia. In case 6, chest X-ray showed diffuse, bilateral ground-glass and interstitial opacities, and pulmonary consolidations. The gross examination showed diffuse lung parenchyma consolidation (formalin-fixed lung, red arrow). At histology, multiple foci of diffuse alveolar damage and organizing pneumonia were seen (black arrow) with squamous metaplasia (black arrowhead). Hematoxylin-eosin, scale bar: 200 μm. Tissue molecular analysis for SARS-CoV-2 was positive. In case 14, chest X-ray demonstrated ground-glass opacities and pleural effusion. The gross examination showed a grayish granular and friable area in the lung (red arrowhead). At histology areas of granulocyte, infiltration with abscess-like features was seen (bacterial pneumonia, black arrow). Hematoxylin-eosin, scale bar: 300 μm. Tissue molecular analysis for SARS-CoV-2 was positive. In case 12, chest X-ray showed signs of pulmonary vascular congestion, interstitial thickening, ground-glass opacities, bilateral pulmonary consolidations, and pleural effusion. The gross examination showed nodular lymphangitis and lymph node metastasis (red arrow). At histology multiple foci of neoplastic thrombi of squamous cell carcinoma were seen (black star). Hematoxylin-eosin, scale bar: 300 μm. Tissue molecular analysis for SARS-CoV-2 was positive. In case 4, chest X-ray demonstrated bilateral signs of pulmonary vascular congestion and ground-glass opacities in the left lung. The gross examination showed the lung with a greenish/brown appearance (formalin-fixed lung). At histology, edema (black arrow) and diffuse infiltration of macrophages with intracytoplasmic bile pigment granules (black arrowhead) were detected. Hematoxylin eosin, scale bar: 200 μm. Tissue molecular analysis for SARS-CoV-2 was negative. ΔRn, the normalized reporter value (Rn) of the experimental reaction minus the Rn value of the baseline signal generated by the instrument; RP, human RNase P gene; N1, region of virus nucleocapsid; N2, region of virus nucleocapsid.

Additional data derived from examination of the entire lungs and other organs allowed us to finally reach a shared confident diagnosis in all cases. In two cases (8%), COVID-19 pneumonia was ruled out, while in three cases (11%), COVID-19 pneumonia was only a marginal pathological process compared to other pathological lesions (Table 2). Examples of definite, probable, possible, and non-COVID-19 pneumonia are given in Figure 2.

The comparison between the diagnosis made by each specialist and the diagnosis made by the team following discussion and with the availability of the autopsy data showed that the ability to formulate a correct diagnosis increased strikingly from a non-pulmonologist to expert specialists, becoming progressively more accurate at different steps (Table 4).

**Table 4:** Right and wrong diagnoses by each individual specialist and their combination.

	**Overall**	**95% CI**
**Non-specialist**		
Wrong	16 (59.3%)	
Right	11 (40.7%)	24.5–59.3%
**Pulmonologist**		
Wrong	7 (25.9%)	
Right	20 (74.1%)	55.3–86.2%
**Radiologist**		
Wrong	6 (23.1%)	
Right	20 (76.9%)	57.9–88.9%
**Pathologist**		
Wrong	2 (7.4%)	
Right	25 (92.6%)	76.6–97.9%
**Pulmonologist + radiologist**		
Wrong	5 (19.2%)	
Right	21 (80.8%)	62.1–91.5%
**Pulmonologist + radiologist + pathologist**		
Wrong	1 (3.8%)	
Right	25 (96.2%)	81.1–99.8%

*CI, confidence interval*.

**Figure 3:**
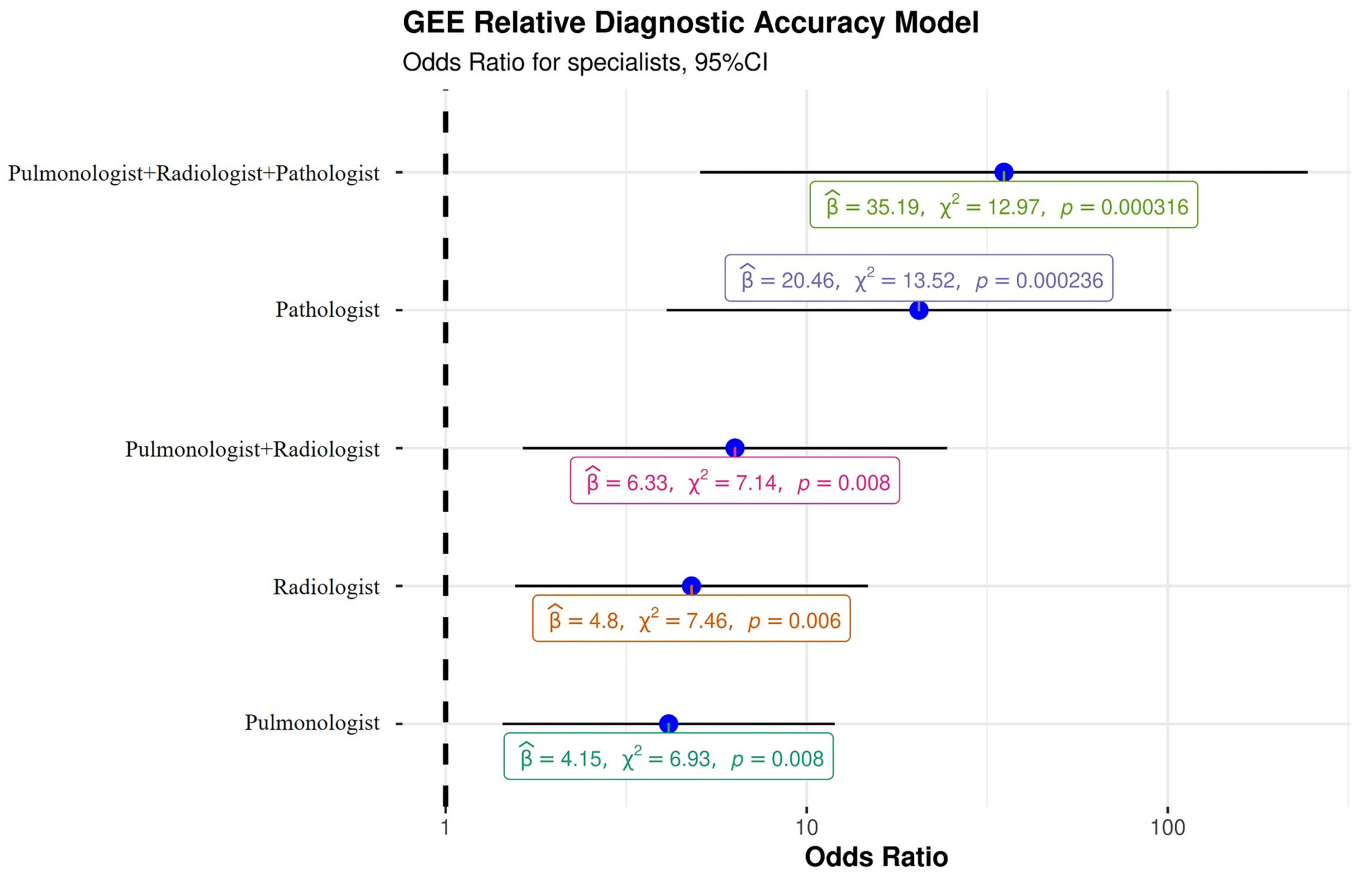
Generalized estimating equation representing the probability of a correct diagnosis for single specialists and for the multidisciplinary team. β, beta, “estimate”; χ2, chi-squared, “regression coefficient.”

The GEE model showed that every single specialist was able to make a significantly more accurate diagnosis than a non-pulmonologist. Hence, a radiologist and pulmonologist were able to make a diagnosis about six times more accurate than a non-pulmonologist [OR: 6.33 (95% CI: 1.63–24.5); *p* = 0.0075]. Of note, the highest level of accuracy was achieved by the combination of three expert specialists (*p* = 0.0003) who made diagnoses that were about 35 times more accurate [OR: 35.2 (95% CI: 5.07–244)] than a single non-pulmonologist (Table 5, Figure 3). Indeed, in only one case (4%, case number 10) the multidisciplinary team diagnosis was wrong when compared to the gold standard (full autopsy diagnosis) (Table 5, Figure 3). In contrast, the diagnosis formulated by a non-pulmonologist was incorrect in over half of the cases (59%), whereas, the diagnosis formulated by a pulmonologist or a thoracic radiologist was not correct in seven (26%) and six cases (23%), respectively. After discussion, radiologists and pulmonologists incorrectly diagnosed five cases (19%). After a full autopsy and whole lung examination, pathologists misinterpreted two cases (7%).

**Table 5:** GEE model for estimating the relative correctness of specialists in respect to the non-specialist diagnosis.

	**Estimate**	**Standard error**	***p*-value**	**95% CI**
Pulmonologist	4.14	0.540	0.0084	1.44–12.0
Radiologist	4.80	0.574	0.0063	1.56–14.8
Pathologist	20.5	0.821	0.0002	4.09–102
Pulmonologist + radiologist	6.33	0.691	0.0075	1.63–24.5
Pulmonologist + radiologist + pathologist	35.2	0.989	0.0003	5.07–244

*CI, confidence interval*.

## Discussion

In this study, we showed that the diagnostic accuracy of a multidisciplinary approach involving dedicated DPLD physicians is significantly higher than that of non-pulmonologists in a subset of patients infected by SARS-CoV-2 who died at the University Hospital of Padova and who underwent autopsy. We demonstrated that the dynamic interaction among DPLD experts influenced the level of confidence for the final diagnosis, which improved step by step. In two cases (8%), the diagnosis of COVID-19-related death was incorrect (final diagnosis: no COVID-19 pneumonia), while in three cases (11%), COVID-19 pneumonia was only a marginal feature compared to other pathological lesions. Thus, in 19% patients the diagnosis was mainly incorrect with consequent inappropriate management. This was the case in two patients, one with severe aspiration pneumonia and the other with carcinomatous lymphangitis who would have required different monitoring and management of care. Inappropriate treatment might have impacted on patient survival and outcome.

The global spread of the SARS-CoV-2 infection was quite unexpected, rapidly leading to a worldwide health emergency. As with any pandemic, patient care has been affected by staffing shortages, a chaotic work environment, and high levels of clinician stress. Clinicians had no choice but to provide care in an extraordinary setting. Moreover, the ICUs rapidly became saturated, and their overcrowding led to the recruitment of non-specialist medical staff, potentially exposing critically ill patients to mismanagement. Based on this distressing experience, COVID-19 health care should be planned adequately during the current second global wave. Today, the challenge is to establish a correct diagnosis taking into consideration several pathological conditions that may mimic and/or overlap with COVID-19 pneumonia, with the aim of optimizing patient management and, consequently, reducing mortality. Although our study consisted of a retrospective analysis (i.e., “a backward path by an expert team”), we believe a multidisciplinary approach involving specialists with experience in DPLD diagnosis and management can be highly beneficial to patient care. The multidisciplinary evaluation has become the diagnostic gold standard for DPLD, as it improves diagnostic confidence and interobserver agreement compared to individual components of the multidisciplinary team in isolation (18, 23), as was the case in our study.

An expert team should be involved in patient evaluation at the very time of hospital admission, particularly when patients are fragile and have severe respiratory failure. The chaotic work environment and the stressful conditions of emergency medical staff, which may make a face-to-face multidisciplinary approach non-realistic, might be successfully overcome by using newer digital technologies. Indeed, during the COVID-19 pandemic, multi-specialist meetings have been suspended and converted into virtual meetings, as occurred in our case.

In the multidisciplinary team of DPLD specialists, radiologists play a key role in that HRCT is largely recognized as a very sensitive and highly specific tool (23–25). During the early phase of the pandemic, CT was seldom performed in COVID-19-positive patients for safety reasons (26, 27). Although chest X-ray proved to be an accurate and reliable method to assess patients with COVID-19, even allowing the development of dedicated scores [CARE referral score] (27), CT plays a crucial role in recognizing alternative diagnoses, especially in patients with pre-existing pulmonary diseases (28–30). Moreover, as recently demonstrated by Borakati and colleagues, it has a higher sensitivity for COVID-19 and its use should be especially promoted in the initial assessment of suspected cases (31). The use of a diagnostic modality other than the gold standard may account for the higher agreement between pathologists and pulmonologists than radiologists in cases of partial agreement. Learning from the difficulties encountered in the first wave of the pandemic, most hospitals worldwide have recently adopted organizational models, which guarantee safe pathways to CT scanners that will surely increase the use of this technique and are expected to have a significant impact on the quality of the delivered care (32, 33).

After discussion, pulmonologists and radiologists achieved a correct diagnosis in 81% of cases and were about six times more accurate than a single non-pulmonologist emphasizing their acceptable diagnostic yield even when histology is not available. These findings are of particular importance since, in some patients with COVID-19 pneumonia, severe respiratory failure could hamper invasive procedures (such as bronchoscopy) and consequently limit the pathologist's contribution. However, we then demonstrated that radiologists and pulmonologists incorrectly diagnosed five cases (19%) which could have influenced the decision-making process inducing a different treatment approach, monitoring, and setting of care. Indeed, in the scenario of an overcrowded intensive care unit, extra effort should be made to seek additional opinions by MDT to obtain greater confidence in the diagnostic impression and to improve the management and outcome of these patients.

In our study, as expected, pathologists showed the highest level of confidence between the first diagnostic impression on a single lung fragment compared to the final diagnosis on whole lung examination, with an incorrect diagnosis being made in only two cases. The lung fragments used by pathologists to perform the first diagnosis were similar in size to those obtained by video-assisted thoracic surgery (VATS) that is suggested to be the gold standard tool for the histological diagnosis of DPLD/ILD (23). Invasive procedures such as VATS carry a high risk of mortality, particularly in patients with severe respiratory dysfunction and under mechanical ventilation (34). During the SARS-CoV-2 pandemic, invasive diagnostic procedures involving sampling of the lung parenchyma were discouraged. However, given the critically important contribution that pathologists could provide in the diagnosis of COVID-19 pneumonia, minimally invasive procedures, such as transbronchial lung biopsy/cryobiopsy could be reconsidered in the diagnostic work-up of COVID-19 pneumonia in doubtful cases, when radiological and/or clinical findings suggest the existence of an additional or alternative pathological condition. This is in line with recent expert recommendations (35) suggesting that bronchoscopy, if opportune, can be safely performed in patients with COVID-19, prioritizing minimization of the risk of viral transmission.

Information coming from a full autopsy of COVID-19 patients with the evaluation of numerous lung samples was considered the gold standard for final diagnosis in our case series. Data provided by the most recent autopsy studies have been crucial in improving our knowledge of the pathological substrates of COVID-19. Indeed, because of the contribution of autopsy studies, COVID-19 pneumonia is now recognized as a complex disease involving not only the lung parenchyma but also the vascular compartment with features that include vasculitis, angiogenesis, capillaritis, and micro/macrothrombi (10, 14, 36).

The present study has several limitations. First, the study is monocentric, and the study population is relatively small. However, despite this, we were able to implement a GEE model that is robust and provides reliable results even with small sample sizes. Moreover, this is one of the largest monocentric European case series wherein the same lung sampling methodology and analysis was consistently applied.

A multidisciplinary approach to diagnosis and management of patients with COVID-19 requires extra effort by the healthcare providers involved but, if it should be validated, it would have the potential to consistently improve the outcome of this often-fatal disease.

## Data Availability Statement

The raw data supporting the conclusions of this article will be made available by the authors, without undue reservation.

## Ethics Statement

The studies involving human participants were reviewed and approved by The Local Ethics Committee of the University Hospital of Padova (4853/A0/20). The patients/participants provided their written informed consent to participate in this study.

## Author Contributions

FC, EB, and PS: conceptualization, writing—reviewing and editing, and supervision. FP, FF, and CG: writing original draft—preparation, visualization, and investigation. FL, EC, LV, SV, CD, and AF: resources and investigation. DG and AC: resources, investigation, visualization, and supervision. All authors have wrote, read, and approved the final version of the manuscript.

## Conflict of Interest

The authors declare that the research was conducted in the absence of any commercial or financial relationships that could be construed as a potential conflict of interest.
